# The validity and reliability of the Swedish version of the Satisfaction with appearance scale for individuals with systemic sclerosis

**DOI:** 10.1177/23971983221107858

**Published:** 2022-07-17

**Authors:** Malin Mattsson, Roger Hesselstrand, Karin Gunnarsson, Elisabet Dyrsmeds, Monica Holmner, Annica Nordin, Carina Boström

**Affiliations:** 1 Department of Neurobiology, Care Sciences and Society, Karolinska Institutet, Stockholm, Sweden; 2 Department of Physiotherapy, Sunderby Hospital, Luleå, Sweden; 3 Department of Clinical Sciences, Lund University, Lund, Sweden; 4 Department of Rheumatology, Skåne University Hospital, Lund, Sweden; 5 Division of Rheumatology, Department of Medicine Solna, Karolinska Institutet and Karolinska University Hospital, Stockholm, Sweden; 6 Division of Rheumatology, Karolinska University Hospital, Stockholm, Sweden; 7 The Swedish Rheumatism Association National Association for Systemic Sclerosis, Stockholm, Sweden; 8 Women’s Health and Health Professionals Theme, Medical Unit Occupational Therapy and Physiotherapy, Karolinska University Hospital, Stockholm, Sweden

**Keywords:** Body image, systemic sclerosis, reliability, validity, questionnaire, patient-reported outcome measure

## Abstract

**Background:**

Systemic sclerosis (SSc) can lead to visible changes in appearance which could generate concerns among patients. Thus, valid questionnaires that capture these concerns are valuable to identify and communicate appearance concerns.

**Objective:**

To determine aspects of the validity and reliability of the Swedish version of the Satisfaction with Appearance scale for individuals with SSc (SWAP-Swe in SSc).

**Methods:**

Content validity was assessed by interviews. In a cross-sectional design, construct validity was evaluated by comparing the self-reported questionnaire SWAP-Swe in SSc to the Scleroderma Health Assessment Questionnaire (SSc HAQ), Patient Health Questionnaire-8 (PHQ-8), RAND-36, modified Rodnan skin score (mRSS), disease duration and age using Spearman’s rank correlations (*r_s_*). Internal consistency was evaluated by Cronbach’s alpha coefficient and corrected item-to-total correlations. Test–retest reliability was investigated using the intraclass correlation coefficient (ICC).

**Results:**

Eleven patients and 10 health professionals participated in the assessment of content validity. For the other aspects of validity and reliability 134 patients (median age 62 years, women 81%, limited cutaneous SSc 75%) participated. Overall, the content validity was satisfactory. The SWAP-Swe in SSc correlated with SSc HAQ (HAQ-DI *r_s_* = 0.50, visual analogue scales *r_s_* = 0.24–0.41), PHQ-8 (*r_s_* = 0.46), RAND-36 (*r_s_* = −0.21 to −0.47), mRSS (*r_s_* = 0.28), disease duration (*r_s_* = −0.01) and age (*r_s_* = −0.15). The Cronbach’s alpha coefficient was 0.92, corrected item-to-total correlations ⩾ 0.45 and the ICC 0.82.

**Conclusion:**

The SWAP-Swe in SSc showed satisfactory content validity, sufficient and good internal consistency and sufficient test–retest reliability. It was more strongly associated with self-reported questionnaires than with physician-assessed skin involvement and age, indicating that appearance concerns in SSc seem to be multidimensional as earlier reported. Our study contributes with a thorough investigation of validity and reliability including aspects that have not been investigated before. However, evaluation of more validity aspects of the SWAP-Swe in SSc is suggested.

## Introduction

Systemic sclerosis (SSc) is a rare autoimmune inflammatory disease, characterised by vasculopathy, fibrosis in the skin and internal organs and often divided into diffuse cutaneous (dcSSc) or limited cutaneous SSc (lcSSc).^1^ The disease often leads to skin thickening and hardening, pigment changes, cutaneous telangiectasia and Raynaud’s phenomenon, changes that commonly involve the face and hands.^2^ Appearance concerns are more frequent among persons with SSc than persons from the general population.^3^ In SSc, changes in appearance in visible body parts such as face and hands are described to be related to body image distress including dissatisfaction with appearance,^4,5^ decreased self-esteem, symptoms of depression and anxiety.^5^ Body image dissatisfaction has been reported to have moderate^6^ associations with symptoms of depression^7,8^ and mental health-related quality of life (HRQL).^8,9^ Furthermore, the association between body image dissatisfaction and disability has been presented as weak to moderate,^7,9^ and the association to skin disease severity has been reported as weak.^9^ To improve quality in life, it is important for the patients to develop coping strategies concerning, for example, body image distress.^10^

As health professionals struggle to support patients with SSc in self-management, including appearance concerns, it is important to have tools to assess body image dissatisfaction. A patient-reported outcome measure (PROM) that assesses social discomfort and dissatisfaction with appearance is the Satisfaction with Appearance (SWAP) scale. The reliability and validity of the SWAP have been evaluated in SSc.^8,9,11^ The SWAP was originally developed for people with burns^12^ and has been adapted for SSc by changing the word ‘burn’ to ‘illness’^13^ or ‘scleroderma’.^8^ Psychometric evaluations of the SWAP have been conducted in the English and French language among persons with SSc, but to date, the Swedish version of the SWAP has not been evaluated in SSc. Validating PROMs in different languages is important to further understand appearance concerns in SSc in different populations and to develop interventions for coping with appearance concerns. Thus, this study aimed to determine aspects of the validity and reliability of the Swedish version of the Satisfaction with Appearance scale for individuals with SSc (SWAP-Swe in SSc).

## Methods

In the first step, the SWAP-Swe in SSc was assessed for content validity. Thereafter, construct validity, internal consistency as well as floor and ceiling effects were evaluated in a cross-sectional design. Finally, test–retest reliability was evaluated.

### Participants

Patients with SSc were recruited from three rheumatology centres in Sweden (Karolinska University Hospital, Skåne University Hospital and Sunderby Hospital). All patients included met the 2013 ACR/EULAR criteria for SSc,^14^ were at least 18 years of age, with disease duration ⩾ 1 year, and could understand and speak Swedish. Patients who were unable to answer written questions were excluded. Patients were enrolled by a rheumatologist during their visit to the rheumatology centre. A convenience sample of patients (n = 11) from one centre and health professionals (HPs) (n = 10) from the two other centres was used to assess content validity. A foremost consecutive sample of patients (n = 134) from two centres participated in the assessment of construct validity, reliability, and floor and ceiling effects. One patient was excluded in this part due to a misunderstanding of the SWAP-Swe in SSc. These sample sizes can be considered very good.^15^ The content validity data were collected between September 2017 and January 2018. For the other aspects of validity and reliability, data were collected between February 2019 and April 2020. Informed written consent was obtained from all participants in accordance with the Helsinki Declaration and the regional ethics committee in Umeå (No. 2017/149-31) approved the study.

### Sociodemographic data and outcome measures

Most patients answered questions about sociodemographic data and completed PROMs in conjunction with their visits to the rheumatology centre. The following PROMs were self-reported using paper and pencil:

The SWAP in SSc consists of 14 items which can be summed into a total score (score range 0–84) or two subscale scores, social discomfort subscale (score range 0–36) and dissatisfaction with appearance subscale (score range 0–48). Items in the dissatisfaction with appearance subscale are reverse scored. Higher scores indicate greater social discomfort and dissatisfaction with appearance.^11^ The SWAP in English has support for reliability and validity in SSc.^8,9,11^

The Scleroderma Health Assessment Questionnaire (SSc HAQ) evaluates disability, pain and disease interference with daily activities.^16^ The SSc HAQ includes the HAQ-Disability Index (HAQ-DI) assessing daily activities (the total score ranges from 0 to 3). Furthermore, the patient rates pain on a 15-cm line visual analogue scale (VAS, 1 cm = 0.2 points, score range from 0 to 3). The SSc HAQ adds five 15 cm VAS evaluating disease interference with daily activities of gastrointestinal symptoms, lung symptoms (breading problems), Raynaud’s phenomenon, digital ulcers and overall disease severity. Scores are calculated for each VAS (score range from 0 to 3). The higher the values, the worse disability, pain and interference with daily activities, respectively. The Swedish SSc HAQ has support for reliability and validity in SSc.^17^

The Patient Health Questionnaire-8 (PHQ-8) assesses depressive symptoms with a total score ranging from 0 to 24 where a higher score represents more depressive symptoms.^18^ The PHQ-8 in Swedish has support for reliability and validity in SSc.^19^

The RAND-36 item (RAND-36) Health Survey assessing HRQL. The RAND-36 is composed of eight subscales ranging from 0 to 100 where a higher score represents a higher level of HRQL. The Swedish RAND-36 is reliable and aspects of validity have been proven.^20^

The physician provided disease-related variables. Skin involvement was assessed by the modified Rodnan skin score (mRSS), where skin thickness is assessed in 17 body areas. The total score can range from 0 to 51; a higher score illustrates greater skin involvement.^21^

### Content validity

A SWAP in the Swedish language has been culturally adapted and validated for persons with burns.^22^ In that version, the English word *chest* was changed to a Swedish word for *torso* and, in another item, the word *interfered* was changed to a Swedish word for *affected/influenced* and the instructions were clarified.^22^ In our study, the word *burn* was changed to a Swedish word for *systemic sclerosis*, and we followed the order of the items in the SWAP version presented by Jewett et al.^11^

Persons with SSc and HPs were interviewed to evaluate content validity.^15^ A pilot interview was conducted with one HP to test and refine the semi-structured interview guide (Table 1). Before the individual interviews, patients completed the SWAP-Swe in SSc (first version) and HPs reflected on the questionnaire. The interviews were sound-recorded, verbatim transcribed and analysed with qualitative content analysis^23^ using a deductive approach.^24^ The text was divided into meaning units, which were coded^23^ and sorted into the following domains: comprehensibility, relevance and comprehensiveness.^15^

**Table 1.** The interview guide covered the following main questions to assess content validity.

What do you think about the comprehensibility of the items? Are any items difficult to understand? How do you experience the introduction and response set? Are there any items you want to exclude/include? Do the items cover relevant aspects of satisfaction with appearance in SSc? *To elaborate the answers, follow-up or clarifying questions were used*

Linguistic and layout adjustments of the SWAP-Swe in SSc were made in response to the results of the interviews after discussions within the research team and the patient research partners. Thereafter, a back-translation to English was performed by a professional translator (native English speaker) to assess correspondence with the English SWAP for individuals with SSc.

### Construct validity, reliability and floor and ceiling effects

After linguistic and layout adjustments of the SWAP-Swe in SSc were made, evaluation of construct validity by the assessment of possible associations between the SWAP-Swe in SSc and other outcomes measures was done.^15^ Reliability was assessed through internal consistency and test–retest reliability and floor and ceiling effects were also evaluated. To evaluate construct validity, internal consistency and floor and ceiling effects data were collected when patients visited the rheumatology centre for enrolment. To assess its test–retest reliability, the SWAP-Swe in SSc was completed a second time at the patient’s home and the answers were returned by mail in a pre-stamped envelope. The mean time between the test occasions was 11 (SD ± 8.1) days.

### Statistical analysis

Construct validity was assessed by Spearman’s rank correlation coefficient (*r_s_*) which was interpreted accordingly: 0 = indicated ‘none’, 0.1–0.3 = ‘weak’, 0.4–0.6 = ‘moderate’, 0.7–0.9 = ‘strong’ and 1.0 = ‘perfect’.^6^ Based on previous results of the SWAP in SSc^7–9^ and clinical assumptions the following was anticipated: The total score of the SWAP-Swe in SSc was expected to correlate ‘moderately’ and positively to depressive symptoms (PHQ-8) and negatively to HRQL (especially mental aspects, RAND-36). ‘Weak’ to ‘moderate’ positive association was expected with disability (HAQ-DI). A ‘weak’ or less correlation was expected between SWAP and pain (HAQ-DI VAS, RAND-36), disease interference with daily activities (SSc HAQ VAS), skin involvement (mRSS), disease duration and age.

Internal consistency was assessed by Cronbach’s alpha coefficient and corrected item-to-total correlation. An alpha coefficient ⩾ 0.70 was considered to be ‘sufficient’^25^ and item correlations > 0.30 as ‘good’.^26^ Intraclass correlation coefficient (ICC) with a two-way-mixed model and absolute agreement^27^ and weighted kappa with quadric weights^28^ were used to evaluate test–retest reliability. The ICC was used for the total score and subscale scores, an ICC ⩾ 0.70 was evaluated as ‘sufficient’.^25^ Weighted kappa were calculated for the items, and values: <0.00 was classified as ‘poor’, 0.00–0.20 = ‘slight’, 0.21–0.40 = ‘fair’, 0.41–0.60 = ‘moderate’, 0.61–0.80 = ‘substantial’ and 0.81–1.00 = ‘almost perfect’.^29^ The sign test was used to assess whether there were any significant changes in the test–retest procedure for the total score, subscales and each item.

Floor and ceiling effects were determined by the numbers of patients scoring the lowest or highest possible score in the total score and subscales and were defined as cases in which > 15% of the patients achieved the lowest or highest possible score.^30^

The level of significance was specified at *p* ⩽ .05. The SPSS, version 26, was used for statistical analyses except for Kappa, which was accomplished using VassarSats: Website for Statistical Computation.

## Results

### Participants

Eleven patients were included in the content validity part, and in the other validly and reliability parts, 134 patients were included and most of them had lcSSc (Table 2). The patients in the first part, for content validity (n = 11) had more extensive skin disease (median mRSS 14 and 36% dcSSc). The second part, for validity and reliability (n = 134) included patients with milder skin disease (median mRSS 2 and 25% dcSSc). Both parts, however, represent a prevalent population with a mean disease duration of 11 years (IQR 6–18 years), respectively. HPs are also described in Table 2.

**Table 2.** Characteristics of health professionals and patients with systemic sclerosis (SSc).

	Content validity (n = 10)^a^ Health professionals	Content validity (n = 11)^b^ Patients with SSc	Construct validity, reliability, floor/ceiling effects (n = 134) Patients with SSc
*Sociodemographic data*			
Women, numbers of patients (%)	8 (80)	10 (91)	108 (81)
Age, years, median (IQR)	56 (43–63)	60 (48–68)	62 (53–71)
Professional experience of SSc care, years, median (IQR)	6 (4–15)		
Married or living together, numbers of patients (%)^†^		8 (73)	88 (66)
College or university, numbers of patients (%)^†^		5 (46)	62 (46)
Professional status, numbers of patients (%)^†^			
Working full or part time		6 (56)	52 (39)
Student or other		0 (0)	4 (3)
Sick listed full or part time		1 (9)	11 (8)
Early retirement full or part time/retired		3 (27)/ 3 (27)	24 (18)/56 (42)
*Disease-related variables*			
Disease duration^c^, years, median (IQR)		11 (6–18)	11 (6–18)
Limited/diffuse cutaneous SSc, numbers of patients (%)		7 (64)/4 (36)	100 (75)/34 (25)
mRSS, score 0–51, median (IQR), min–max		14 (6–26), 4–31	2 (0–4.3), 0–29
Digital ulcers, number of patients (%)		3 (27)	11 (8)
Pitting scars, number of patients (%)		4 (36)	60 (45)
Telangiectasia, number of patients, face/body (%)			83 (62)/89 (66)
*Patient-reported outcome measures*			
SSc HAQ, score 0–3, median (IQR)			
HAQ-DI^†^			.38 (.00–1.00)
HAQ VAS pain^‡^			.48 (.06–1.31)
SSc HAQ VAS^#^			
Gastrointestinal symptoms			.28 (.02–1.14)
Lung symptoms			.26 (.02–.96)
Raynaud’s phenomenon			.52 (.10–1.17)
Digital ulcers			.04 (.00–.64)
Overall disease severity			.67 (.22–1.44)
PHQ-8, score 0–24, median (IQR)^†^			4 (2–8)
RAND-36, score 0–100, median (IQR)^†^			
Physical function			67 (42–85)
Physical role function			50 (0–100)
Bodily pain			68 (45–90)
General health			50 (35–70)
Vitality			55 (36.3–75)
Social function			75 (63–100)
Emotional role function			67 (33–100)
Mental health			72 (60–88)

IQR: interquartile range; HAQ-DI: Health Assessment Questionnaire-Disability Index; mRSS: Modified Rodnan Skin Score; PHQ-8: Patient Health Questionnaire-8; RAND-36: RAND-36 item Health Survey; SSc HAQ: Scleroderma Health Assessment Questionnaire; VAS: visual analogue scale.

a Two from each of the following professions: nurse, occupational therapist, physicians, physiotherapist and social worker.

b Patients not included in test of construct validity, reliability and floor/ceiling effects.

c Disease duration referred to the time from the first non-Raynaud’s symptom.

† One to three missing values in the n = 134 sample; ^‡^18 missing values in the n = 134 samples; ^#^8 to 10 missing values in the n = 134 samples.

Missing items were treated accordingly to the instructions of a respective questionnaire.

### Content validity

The analysed interviews showed that the SWAP-Swe in SSc was in general experienced easy to comprehend, items were overall relevant and covered key aspects of satisfaction of appearance (Table 3). From the results of the interviews, the following main changes were done. To support the understanding of the response set, the number 0 to 6 was added below each item with the anchor words (0 = strongly disagree, 6 = strongly agree). Furthermore, items 4, 5 and 6 were clarified that they referred to changes in appearance caused by SSc. In item 8, the word *hair* was added to increase the relevance of this item (i.e. scalp/hair), and in item 14, the word *upper body* was added (i.e. torso/upper body) to ease the understanding.

**Table 3.** Content validity of the Swedish version of the Satisfaction with appearance scale for individuals with systemic sclerosis (SWAP-Swe in SSc).

Domains	Results from the analysis of interviews of persons with SSc (patients) and healthcare professionals (HPs), and examples of quotes
Comprehensibility	In general, the instruction and response options were easy to follow and items were experienced as clear. However, some found the response options as extensive and HPs expressed that it could be difficult to separate the response options ‘strongly agree’ from ‘agree’ and ‘strongly disagree’ from ‘disagree’. Among patients, the reverse scoring related to the dissatisfaction of appearance subscale was experienced as supportive to diminish risk of un-reflected responses, but others found the reversed scoring as unclear which also influenced their responses. Patients and HPs expressed that it could be difficult to determine which persons in the social environment the items designated, for example ‘others’ in item 5 and ‘relationships’ in item 4. Furthermore, when words such as ‘changes in my appearance caused by my scleroderma’ was absent in items they could be experienced as less clear such as in item 5 and 6 in the social discomfort subscale as well the items related to the Dissatisfaction with appearance subscale. Among patients, the word ‘chest’ in item 14 was experienced as difficult to understand. *‘it could be described in which direction the scale is. . . well, because I had to go back repeatedly . . . to think about. . .what should I answer. . . in which direction is now zero and which direction is six’* (Patient 8) *‘I do not think there is something that I feel is unclear, rather I believe in fact that patients would understand it’* (HP 2)
Relevance	The items were experienced as overall relevant. Some patients experienced disturbing changes in their appearance which they concealed. Others expressed accepting their appearance changes or that SSc had not changed their appearance, thus these patients found the items as more relevant for others than for themselves. It was also expressed that items in the dissatisfaction with appearance subscale were not relevant when not experience appearance changes. Both patients and HPs reflected on the necessity of item 8 (. . .the appearance of my scalp) as they did not experience that the scalp was affected, or if not bald not visible. Emotional distress could be associated with appearance concerns, for example items 6 (‘I don’t think people would want to touch me’). HPs expressed that the SWAP-Swe in SSc could be emotional demanding for patients and they feared hurt reactions. Among HPs it was expressed as inappropriate to focus on patients’ appearance and they feared to imply that there were anything wrong with patients’ appearance, for example by items in the Social discomfort subscale. Nevertheless, HPs experienced that patients could have appearance concerns and they wanted to support patients’ needs. Thoughts were expressed about when and how to use the questionnaire, in appearance concerns or only in patients who were confident with the disease and its consequences. ‘*I’m so slightly affected, so I do not feel, absolutely not with my family and my grandchildren they do not distance themselves from me, they are close to me and they have never asked anything,. . .and not together with friends either. However, maybe when I meet new people I feel that. . .they might wonder, why she looks like that, or maybe I do not feel so comfortable then’* (Patient 7) ‘*Sometime patients say that they find it* (appearance) *difficult. . . if they have a lot of telangiectasia in the face. . . but it is not something that is focused on very much in healthcare, at the same time as it is certainly something that patients focus a lot on, because it is difficult’* (HP 9)
Comprehensiveness	Overall items were experienced to cover important aspects of satisfaction of appearance in SSc. The whole body was in general covered by the items making dissatisfaction possible to capture by the items. However, items concerning appearance of mouth, lips, nose, fingers, nails, and feet were suggested to be included. Moreover, HPs experienced that other aspects of appearance such as stiffness and limping when moving or worries of changes of appearance in the future were lacking. *‘I would suggest adding* (an item) *about teeth. . . because when you have this disease it reduces the saliva, so the teeth becomes bad or you lose teeth’* (Patient 2) *‘You have probably got everything included for this* (appearance), *even the scalp is included, so I mean you have probably caught it. . . and got everything. . . as you can imagine’* (HP 6)

### Construct validity

‘Moderate’ correlations were found between the SWAP-Swe in SSc total score and disability, pain/bodily pain, disease interference with daily activities of gastrointestinal symptoms and overall disease severity, depressive symptoms, general health, vitality, social function and mental health. ‘Weak’ correlations were found with disease interference with daily activities of lung symptoms, Raynaud’s phenomenon and digital ulcers, physical function, physical role function and emotional role function, skin involvement and age (Table 4).

**Table 4.** Construct validity (correlations) of the Swedish version of the Satisfaction with appearance scale for individuals with systemic sclerosis (SWAP-Swe in SSc).

		The SWAP-Swe in SSc total score (n = 112–128)^#^ *r_s_*	*p*
*Disability, pain and disease interference with daily activities*, SSc HAQ			
HAQ-DI		0.50	<0.001
HAQ-DI VAS	Pain	0.41	<0.001
SSc HAQ VAS	Gastrointestinal symptoms	0.39	<0.001
	Lung symptoms	0.24	0.009
	Raynaud’s phenomenon	0.31	<0.001
	Digital ulcers	0.29	<0.001
	Overall disease severity	0.41	<0.001
*Depressive symptoms*, PHQ-8		0.46	<0.001
*Quality of life*, RAND-36			
Physical function		−0.34	<0.001
Physical role function		−0.33	<0.001
Bodily pain		−0.42	<0.001
General health		−0.38	<0.001
Vitality		−0.47	<0.001
Social function		−0.47	<0.001
Emotional role function		−0.22	0.013
Mental health		−0.39	<0.001
*Skin involvement*, mRSS		0.28	0.001
*Disease duration*		−0.04	0.679
*Age*		−0.15	0.091

*r_s_*: Spearman rank correlation coefficient; HAQ-DI: Health Assessment Questionnaire-Disability Index; mRSS: modified Rodnan skin score; PHQ-8: Patient health questionnaire-8; RAND-36: RAND 36-Item Health Survey; SSc HAQ: Scleroderma Health Assessment Questionnaire; VAS: visual analogue scale.

The SWAP-Swe in SSc total score correlated *r_s_* = 0.69 (*p* < .001) with the social discomfort subscale and *r_s_* = 0.94 (*p* < .001) with the dissatisfaction with appearance subscale.

# Correlations calculated in n = 112–128 due to missing values.

### Reliability, floor and ceiling effects

For the SWAP-Swe in SSc total score and subscales, the Cronbach’s alpha coefficients ranged from 0.90 to 0.93 and corrected item-to-total correlations ranged from 0.45 to 0.86 (Table 5). There were no significant differences between patients participating in the first test and retest occasion in terms of age, disease duration, disease subtype and mRSS (data not reported).

**Table 5.** Internal consistency and test–retest reliability of the Swedish version of the Satisfaction with appearance scale for individuals with systemic sclerosis (SWAP-Swe in SSc).

	Cronbach’s alpha coefficient^a^		Test Median (IQR) (n = 134)^b^	Retest Median (IQR) (n = 124)^b^	Intraclass correlation coefficient	Sign test P
The SWAP-Swe in SSc (0–84 total scores)	0.92		22.5 (11.3–34)^†^ x̄ 24.5 (SD ± 16.2)^†^	23 (10–35)^‡^ x̄ 23.6 (SD ± 16.6)^‡^	0.82	1.00
Social discomfort subscale (0–36 scores)	0.90		3 (0–7)^§^	4 (0–8)	0.83	0.90
Dissatisfaction with appearance subscale (0–48 scores)	0.93		18 (10–27.3)^§§^	18 (9–25)‡	0.71	0.84
In each of the following statements, circle the most correct responses for you according to this scale: 0 = strongly disagree; 1 = disagree; 2 = somewhat disagree; 3 = neutral; 4 = somewhat agree; 5 = agree; 6 = strongly agree *För vart och ett av nedanstående påståenden (i delskala A och B) ber vi dig ringa in de svar som stämmer bäst in på dig enligt följande skala: 0* *=instämmer inte alls; 1* *=instämmer i mycket liten utsträckning; 2* *=instämmer i liten utsträckning; 3* *=varken eller; 4* *=instämmer i ganska stor utsträckning; 5* *=instämmer i stor utsträckning; 6* *=instämmer helt* Items 1–14 in English and Swedish (0–6 scores)	Corrected item-to-total correlation^a^				Weighted kappa	
	Total score	Subscale scores				
Social discomfort subscale						
1. Because of changes in my appearance caused by my scleroderma, I am uncomfortable in the presence of my family *På grund av förändringar av mitt utseende orsakat av systemisk skleros känner jag mig besvärad när jag är tillsammans med min familj*	0.45	0.63	0 (0–1)^#^	0 (0–1)	0.52	0.04
2. Because of changes in my appearance caused by my scleroderma, I am uncomfortable in the presence of my friends *På grund av förändringar av mitt utseende orsakat av systemisk skleros känner jag mig besvärad när jag är tillsammans med mina vänner*	0.63	0.86	0 (0–2)^#^	0.5 (0–2)	0.77	1.00
3. Because of changes in my appearance caused by my scleroderma, I am uncomfortable in the presence of strangers *På grund av förändringar av mitt utseende orsakat av systemisk skleros känner jag mig besvärad när jag är tillsammans med främmande personer*	0.62	0.84	1 (0–2)^#^	1 (0–2)	0.80	0.60
4. Changes in my appearance have interfered with my relationships *Förändringar av mitt utseende orsakat av systemisk skleros har påverkat mina relationer.*	0.57	0.75	0 (0–1)^#^	0 (0–1)	0.78	0.01
5. I feel that my scleroderma is unattractive to others *Jag har en känsla av att förändringar av mitt utseende orsakat av systemisk skleros är oattraktivt för andra*	0.57	0.79	1 (0–2)^##^	1 (0–2)	0.84	0.88
6. I don’t think people would want to touch me *Jag tror att folk inte skulle vilja röra vid mig på grund av förändringar av mitt utseende orsakat av systemisk skleros*	0.53	0.71	0 (0–1)^##^	0 (0–1)	0.72	0.16
	Corrected item-to-total correlation^a^		Test Median (IQR) (n = 134)^b^	Retest Median (IQR) (n = 124)^b^	Weigthed kappa	Sign test P
Dissatisfaction with appearance subscale						
7. I am satisfied with my overall appearance *Jag är nöjd med mitt utseende totalt sett*	0.74	0.79	2 (1–3)^#^	2 (1–3)	0.63	1.00
8. I am satisfied with the appearance of my scalp *Jag är nöjd med utseendet på min/mitt hårbotten/hår*	0.53	0.65	1 (0–3)^##^	1 (0–3)	0.66	0.37
9. I am satisfied with the appearance of my face *Jag är nöjd med utseendet på mitt ansikte*	0.83	0.84	2 (1–4)^##^	2 (1–4)	0.71	1.00
10. I am satisfied with the appearance of my neck *Jag är nöjd med utseendet på min hals*	0.76	0.80	2 (0–3)	2 (0.3–3)	0.63	0.80
11. I am satisfied with the appearance of my hands *Jag är nöjd med utseendet på mina hander*	0.64	0.59	3.5 (2–5)	3 (1–5)^##^	0.67	0.10
12. I am satisfied with the appearance of my arms *Jag är nöjd med utseendet på mina armar*	0.72	0.79	2 (1–3)	2 (0–3)	0.63	0.90
13. I am satisfied with the appearance of my legs *Jag är nöjd med utseendet på mina ben*	0.57	0.71	2 (1–4)	2 (1–4)	0.68	0.40
14. I am satisfied with the appearance of my chest *Jag är nöjd med utseendet på min bål/överkropp*	0.75	0.84	2 (1–4)	2 (1–3)	0.71	0.01

IQR: interquartile range; x̄: mean; SD: standard deviation.

The English items and subscales of the SWAP are reprinted from Body Image 2017, vol 22, 97–102. Jewett LR, Kwakkenbos L, Hudson M, Baron M, Thombs BD, Canadian Scleroderma Research Group. Assessment of English-French differential item functioning of the Satisfaction with Appearance Scale (SWAP) in systemic sclerosis, with permission from Elsevier, 2022; Permission to use and adjust the SWAP into a Swedish context of SSc was obtained from one of the original developers (personal communication with Professor John Lawrence).

No total score or subscale scores of the SWAP-Swe in SSc were calculated if items were missing.

Higher scores indicate greater social discomfort (item 1–6) and dissatisfaction with appearance (item 7–14).

a Data calculated from the first test occasion in the test–retest procedure.

b On the first test occasion, 134 patients completed the SWAP-Swe in SSc and 124 (93%) on the second occasion.

† Six missing total scores; ^‡^one missing score (total score/subscale score); ^§^two missing subscale scores; ^§§^four missing subscale scores.

# Two missing items; ^##^one missing item.

The ICC for the total score and subscales ranged from 0.71 to 0.83 and the weighted kappa for the items had a median of 0.70 and ranged from 0.52 to 0.84 (Table 5). There were no significant differences in the SWAP-Swe in SSc total score and subscales between the test and retest. Two items (items 1 and 4) were significantly higher upon retest, and one item (item 14) was significantly lower upon retest (Table 5).

There were no floor and ceiling effects for the total score or the subscales except for a floor effect in the social discomfort subscale since 48 (36%) of the patients scored the lowest possible score (score 0).

## Discussion

The SWAP-Swe in SSc was interpreted as having overall satisfactory content validity. When it comes to construct validity, the SWAP-Swe in SSc was overall more strongly associated with self-reported questionnaires concerning disability, depressive symptoms and HRQL than with physicians-assessed skin involvement and age. The internal consistency was classified as ‘sufficient’ and ‘good’ and the test–retest reliability was classified as ‘sufficient’. In addition, there were no floor and ceiling effects, except for a floor effect in the social discomfort subscale.

The results of the analysis of the interviews indicated that the content validity of the SWAP-Swe in SSc was overall satisfactory. However, some patients experienced it as unclear when the response set held a reverse meaning between the subscales as a result of negatively formulated items (e.g. ‘I feel that my scleroderma is unattractive to others’) in contrast to positively stated items (e.g. ‘I am satisfied with the appearance of my face’). Furthermore, among HPs, it was expressed that the items could be emotionally demanding and it was experienced as difficult to know when to use the questionnaire. Thus, a careful introduction of the questionnaire and discussion between the HP and the patient about the completed items would be valuable to diminish any possible misunderstandings or distress. When it comes to when to use the SWAP-Swe in SSc, attention to appearance concerns is of value in SSc^7^ since worries about appearance^31^ and changes of appearance^32^ have been reported among unmet needs for healthcare in patients with SSc.^31,32^ In our study, we described aspects of satisfaction with one’s appearance that were expressed as missing in the SWAP-Swe in SSc, for example, specific parts of the face, hands, feet and limping issues. When needed, these aspects might be subject to additional discussion with the patients when following up on their questionnaire responses.

The construct validity analysis indicated that the majority of the correlations were consistent with our expected hypothesis. Counter to our expectations, we found ‘weak’ correlations between the SWAP-Swe in SSc and HRQL aspects of physical function, physical role function and emotional role function. Mills et al.^9^ also found a ‘weak’ correlation between the SWAP and the physical components of SF-36. Furthermore, counter to our expectation we found a ‘moderate’ correlation instead of a ‘weak’ correlation between the SWAP-Swe in SSc and pain/bodily pain. This discrepancy between our results and results from previous studies might be explained by differences in samples,^9^ pain outcome measures^7,8^ and analysis method.^8,9^ Higher correlations (‘moderate’) than postulated were found between the SWAP-Swe in SSc and disease interference of daily activities of gastrointestinal symptoms and overall disease severity in our study. Although gastrointestinal symptoms are a common problem in SSc,^33^ appearance concerns might influence how the person experiences their disease severity and vice versa. The SWAP-Swe in SSc correlated with other PROMs but also, to some extent, with skin involvement as assessed by a physician as well as age; this highlights that appearance concerns in SSc seem to be multidimensional, as earlier reported.^34^ The associations between appearance concerns and whether the patients have lcSSc or dcSSc might also differ.^34,35^ Additional studies are needed to further understand the associations between the SWAP-Swe in SSc and other disease variables that might affect body appearance (e.g. skin pigmentations and telangiectasia) as well as associations with gender. Taken together, support for construct validity was indicated in the present study.

Internal consistency showed Cronbach’s alpha coefficients that were ‘sufficient’^25^ for the SWAP-Swe in SSc findings that are in agreement with previous reports^8,9,11^ and corrected item-to-total correlations supported ‘good’ internal consistency.^26^ The test–retest reliability for the total score and subscales showed no significant difference between the test occasions. However there were significant differences in three items, which might be explained by the fact that the questionnaire was completed at the hospital and home, respectively, and the latter could give rise to more reflection on appearance issues. The agreements between the test occasions revealed ICCs for the SWAP-Swe in SSc were ‘sufficient’^25^ for the total score and subscales. Heinberg et al.^13^ also found a ‘sufficient’ ICC for the subscales of the SWAP.

We only found a floor effect in the social discomfort subscale. When comparing our findings with a previous SSc study, their social discomfort subscale scores were in absolute values slightly higher,^11^ which indicate less social discomfort in our patients. Appearance concerns in SSc are more common among women,^3^ and younger age is related to higher body image dissatisfaction.^7^ The individuals in our study were in absolute values somewhat older than in some other studies^7,9^ and included both men and women which might contribute to the floor effect in the social discomfort subscale. Furthermore, both subscales in the SWAP are significantly higher in dcSSc,^35^ and in our study, there were more patients with lcSSc. Although there was a floor effect in the social discomfort subscale, some patients indicated distress and the highest social discomfort was reported among the presence of strangers and feeling unattractive to others. When it comes to dissatisfaction with appearance, the appearance with the hands had the highest dissatisfaction.

One methodological limitation in our study is that when assessing construct validity, no other PROMs assessing body image dissatisfaction^36^ was included. Unfortunately, no such PROM was available validated in the Swedish language in SSc. Another possible limitation could be that the recruitment procedure that was a mixture of convenient and consecutive may not have been the most optimal sampling. However, the median age, gender and proportion of lcSSc in the cross-sectional design were similar as described in a population-based study of SSc in Sweden,^37^ which support generalisability of the SWAP-Swe in SSc. Also, the majority of all patients that were included in our study had mild skin involvement; however, it included both moderate and severe skin involvement. Another limitation is that aspects such as skin pigmentations and joint involvements that might influence appearance were not examined. On the other hand, data of digital ulcers, pitting scars and telangiectasia are described.

Even if our study performed a thorough investigation of validity and reliability, future research on the validity, such as structure validity, of the SWAP Swe in SSc would be of value to investigate. Discriminative validity would be beneficial to examine as well, for example, the questionnaire’s ability to discriminate between those with different body appearance involvements. In order to evaluate interventions that focuses on appearance concerns, responsiveness of the SWAP-Swe in SSc needs to be evaluated. Furthermore, feasibility may also be evaluated of the SWAP-Swe in SSc. The Brief-SWAP have in some studies found to be more feasible than its full version with 14 items.^8,15^

In conclusion, the present study indicates that the SWAP-Swe in SSc is overall valid and reliable, and contributes with evaluation of aspect not studied before concerning SWAP. The SWAP-Swe in SSc may be used in research and clinical praxis to identify and communicate appearance concerns among patients. Furthermore, using the questionnaire could give insight into how to support coping strategies regarding appearance concerns. More studies on other aspects of validity of the SWAP-Swe in SSc as well as further research on the questionnaire in patients with dcSSc would be valuable.
